# (*E*)-1-(2,4-Dinitro­phen­yl)-2-[1-(2-nitro­phen­yl)ethyl­idene]hydrazine

**DOI:** 10.1107/S1600536811042620

**Published:** 2011-10-29

**Authors:** Boonlerd Nilwanna, Suchada Chantrapromma, Patcharaporn Jansrisewangwong, Hoong-Kun Fun

**Affiliations:** a Crystal Materials Research Unit, Department of Chemistry, Faculty of Science, Prince of Songkla University, Hat-Yai, Songkhla 90112, Thailand; b X-ray Crystallography Unit, School of Physics, Universiti Sains Malaysia, 11800 USM, Penang, Malaysia

## Abstract

The title compound, C_14_H_11_N_5_O_6_, was obtained from the condensation reaction of 2,4-dinitro­phenyl­hydrazine and 2-nitro­acetophenone. The mol­ecule displays an *E* conformation about the C=N double bond and an intra­molecular N—H⋯O hydrogen bond generates an *S*(6) ring motif. The dihedral angle between the benzene rings is 7.84 (6)°. In the crystal, mol­ecules are linked by C—H⋯O hydrogen bonds and π–π stacking inter­actions [centroid–centroid distance = 3.6447 (8) Å] into a three-dimensional network.

## Related literature

For bond-length data, see: Allen *et al.* (1987 ▶). For hydrogen-bond motifs, see: Bernstein *et al.* (1995 ▶). For related structures, see: Fun *et al.* (2011 ▶); Shan *et al.* (2003 ▶). For background to and the physiological and biological activity of hydro­zones, see: Bendre *et al.* (1998 ▶); Nakamura & Goto (1996 ▶); Rollas & Küçükgüzel (2007 ▶); Singh *et al.* (2005 ▶); Yacorb (1999 ▶). For the stability of the temperature controller used in the data collection, see Cosier & Glazer (1986 ▶).
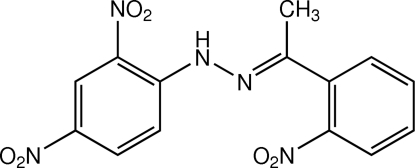


## Experimental

### 

#### Crystal data

C_14_H_11_N_5_O_6_
*M* *_r_* = 345.28
Monoclinic, 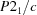

*a* = 11.9313 (9) Å
*b* = 8.6700 (7) Å
*c* = 15.2363 (9) Å
β = 112.455 (5)°
*V* = 1456.61 (19) Å^3^
*Z* = 4
Mo *K*α radiation
μ = 0.13 mm^−1^
*T* = 100 K
0.40 × 0.16 × 0.13 mm

#### Data collection

Bruker APEXII CCD area-detector diffractometer
Absorption correction: multi-scan (*SADABS*; Bruker, 2005 ▶) *T*_min_ = 0.951, *T*_max_ = 0.984
16360 measured reflections
4244 independent reflections
3361 reflections with *I* > 2σ(*I*)
*R*_int_ = 0.033

#### Refinement

*R*[*F* ^2^ > 2σ(*F* ^2^)] = 0.041
*wR*(*F* ^2^) = 0.110
*S* = 1.04
4244 reflections
227 parameters
H-atom parameters constrained
Δρ_max_ = 0.35 e Å^−3^
Δρ_min_ = −0.28 e Å^−3^

### 

Data collection: *APEX2* (Bruker, 2005 ▶); cell refinement: *SAINT* (Bruker, 2005 ▶); data reduction: *SAINT*; program(s) used to solve structure: *SHELXTL* (Sheldrick, 2008 ▶); program(s) used to refine structure: *SHELXTL*; molecular graphics: *SHELXTL*; software used to prepare material for publication: *SHELXTL* and *PLATON* (Spek, 2009 ▶).

## Supplementary Material

Crystal structure: contains datablock(s) global, I. DOI: 10.1107/S1600536811042620/rz2643sup1.cif

Structure factors: contains datablock(s) I. DOI: 10.1107/S1600536811042620/rz2643Isup2.hkl

Supplementary material file. DOI: 10.1107/S1600536811042620/rz2643Isup3.cml

Additional supplementary materials:  crystallographic information; 3D view; checkCIF report

## Figures and Tables

**Table 1:** Hydrogen-bond geometry (Å, °)

*D*—H⋯*A*	*D*—H	H⋯*A*	*D*⋯*A*	*D*—H⋯*A*
N1—H1⋯O2	0.88	1.94	2.6026 (13)	131
C10—H10*A*⋯O4^i^	0.93	2.42	3.2313 (16)	146
C12—H12*A*⋯O4^ii^	0.93	2.55	3.4353 (18)	159

Symmetry codes: (i) 
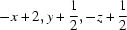
; (ii) 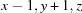.

**Fig. 1.**
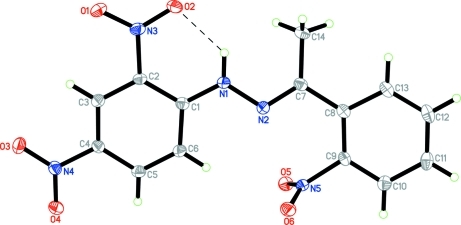
The molecular structure of the title compound, showing 50% probability displacement ellipsoids. Hydrogen bond is shown as a dashed line.

**Fig. 2.**
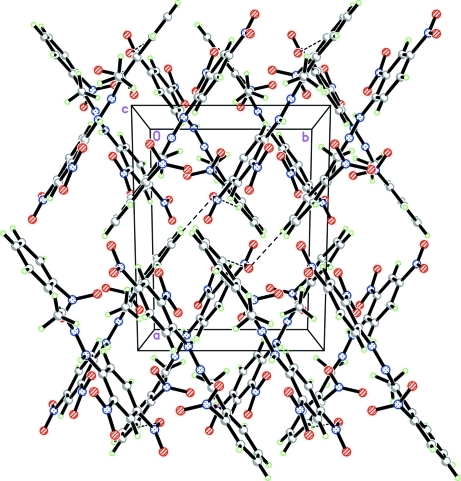
The crystal packing of the title compound viewed along the c axis, showing molecular stacking along the b axis. Hydrogen bonds are shown as dashed lines.

## supplementary crystallographic information

### Comment

Hydrazones exhibit physiological and biological activities in the treatment of several diseases with anticonvulsant, antidepressant, analgesic, antiinflammatory, antiplatelet, antimalarial, antimicrobial, antimycobacterial, antitumor, vasodilator, antiviral, antischistosomiasis (Singh *et al.*, 2005; Rollas & Küçükgüzel, 2007) and tyrosinase inhibitory properties (Bendre *et al.*, 1998). Furthermore, they were used in engineering and analytical studies for aldehydes and ketones sampling (Yacorb, 1999) and analysis of protein carbonyls (Nakamura & Goto, 1996). These interesting activities have led us to synthesize the title hydrazone derivative (I). It was screened for antioxidant and antibacterial activities. Our results found that (I) is inactive for these tests. Herein we report the synthesis and crystal structure of (I).

The whole molecule of (I) (Fig. 1), C_14_H_11_N_5_O_6_, is not planar and exists in an *E* configuration with respect to the ethylidene C═N double bond [1.2905 (15) Å] with the torsion angle N1–N2–C7–C8 = 177.22 (10)°. The dihedral angle between the two benzene rings is 7.84 (6)°. The middle ethylidenehydrazine fragment (C7/C14/N1/N2) is planar with the *r.m.s* deviation of 0.0047 (1) Å. This middle C/C/N/N plane makes the dihedral angles of 11.28 (8) and 9.78 (8)° with the C1–C6 and C8–C13 benzene rings, respectively. The two nitro groups of 2,4-dinitrophenyl are co-planar with the bound benzene ring with the *r.m.s*. deviation of 0.0369 (1) Å for the twelve non H-atoms. However the nitro group of the 2-nitrophenyl tilts away from its bound benzene ring with the dihedral angle of 81.19 (7)° between the C9/N5/O5/O6 plane and C8–C13 benzene ring. This orientation is caused by the steric interaction between the hydrazine and nitro group. An intramolecular N1—H1···O2 hydrogen bond between the hydrazone-NH and the *ortho* nitro group (Fig. 1 and Table 1) generates an S(6) ring motif (Bernstein *et al.*, 1995). The bond distances are within the expected range (Allen *et al.*, 1987) and are comparable with those of related structures (Fun *et al.*, 2011; Shan *et al.*, 2003).

In the crystal structure, the molecules are linked by weak C—H···O hydrogen bonds (Table 1) into a three-dimensional network (Fig. 2) enforced by π···π stacking interactions (*Cg*_1_···*Cg*_2_^i^ = 3.6447 (8) Å; *Cg*_1_ and *Cg*_2_ are the centroids of C1–C6 and C8–C13 benzenre rings, respectively; symmetry code: (i) 2 - *x*, 2 - *y*, 1 - *z*). Short C···O (2.9999 (15) Å] contacts are also observed.

### Experimental

The title compound was synthesized by dissolving 2,4-dinitrophenylhydrazine (0.40 g, 2 mmol) in ethanol (10.00 ml), and H_2_SO_4_ (98%, 0.50 ml) was slowly added with stirring. 2-Nitroacetophenone (0.27 ml, 2 mmol) was then added to the solution with continuous stirring. The solution was refluxed for 1 h yielding a yellow solid, which was filtered off and washed with methanol. Yellow block-shaped single crystals of the title compound suitable for *X*-ray structure determination were recrystalized from ethanol by slow evaporation of the solvent at room temperature over several days. M.p. 443–444 K.

### Refinement

All H atoms were positioned geometrically and allowed to ride on their parent atoms, with d(N—H) = 0.88 Å, d(C—H) = 0.93 Å for aromatic and 0.96 Å for CH_3_ atoms. The *U*_iso_ values were constrained to be 1.5*U*_eq_ of the carrier atom for methyl H atoms and 1.2*U*_eq_ for the remaining H atoms. A rotating group model was used for the methyl groups.

### Crystal data

**Table:**

C_14_H_11_N_5_O_6_	*F*(000) = 712
*M**_r_* = 345.28	*D*_x_ = 1.574 Mg m^−^^3^
Monoclinic, *P*2_1_/*c*	Melting point = 443–444 K
Hall symbol: -P 2ybc	Mo *K*α radiation, λ = 0.71073 Å
*a* = 11.9313 (9) Å	Cell parameters from 4244 reflections
*b* = 8.6700 (7) Å	θ = 1.9–30.0°
*c* = 15.2363 (9) Å	µ = 0.13 mm^−^^1^
β = 112.455 (5)°	*T* = 100 K
*V* = 1456.61 (19) Å^3^	Block, yellow
*Z* = 4	0.40 × 0.16 × 0.13 mm

### Data collection

**Table:**

Bruker APEXII CCD area-detector diffractometer	4244 independent reflections
Radiation source: sealed tube	3361 reflections with *I* > 2σ(*I*)
graphite	*R*_int_ = 0.033
φ and ω scans	θ_max_ = 30.0°, θ_min_ = 1.9°
Absorption correction: multi-scan (*SADABS*; Bruker, 2005)	*h* = −16→16
*T*_min_ = 0.951, *T*_max_ = 0.984	*k* = −9→12
16360 measured reflections	*l* = −21→14

### Refinement

**Table:**

Refinement on *F*^2^	Primary atom site location: structure-invariant direct methods
Least-squares matrix: full	Secondary atom site location: difference Fourier map
*R*[*F*^2^ > 2σ(*F*^2^)] = 0.041	Hydrogen site location: inferred from neighbouring sites
*wR*(*F*^2^) = 0.110	H-atom parameters constrained
*S* = 1.04	*w* = 1/[σ^2^(*F*_o_^2^) + (0.0493*P*)^2^ + 0.498*P*] where *P* = (*F*_o_^2^ + 2*F*_c_^2^)/3
4244 reflections	(Δ/σ)_max_ = 0.001
227 parameters	Δρ_max_ = 0.35 e Å^−^^3^
0 restraints	Δρ_min_ = −0.28 e Å^−^^3^

### Special details

**Table:**

Experimental. The crystal was placed in the cold stream of an Oxford Cryosystems Cobra open-flow nitrogen cryostat (Cosier & Glazer, 1986) operating at 120.0 (1) K.
Geometry. All e.s.d.'s (except the e.s.d. in the dihedral angle between two l.s. planes) are estimated using the full covariance matrix. The cell e.s.d.'s are taken into account individually in the estimation of e.s.d.'s in distances, angles and torsion angles; correlations between e.s.d.'s in cell parameters are only used when they are defined by crystal symmetry. An approximate (isotropic) treatment of cell e.s.d.'s is used for estimating e.s.d.'s involving l.s. planes.
Refinement. Refinement of *F*^2^ against ALL reflections. The weighted *R*-factor *wR* and goodness of fit *S* are based on *F*^2^, conventional *R*-factors *R* are based on *F*, with *F* set to zero for negative *F*^2^. The threshold expression of *F*^2^ > σ(*F*^2^) is used only for calculating *R*-factors(gt) *etc*. and is not relevant to the choice of reflections for refinement. *R*-factors based on *F*^2^ are statistically about twice as large as those based on *F*, and *R*- factors based on ALL data will be even larger.

### Fractional atomic coordinates and isotropic or equivalent isotropic displacement parameters (Å^2^)

**Table:**

	*x*	*y*	*z*	*U*_iso_*/*U*_eq_	
O1	1.32132 (8)	0.59779 (12)	0.77977 (6)	0.0221 (2)	
O2	1.14527 (9)	0.70362 (12)	0.74461 (6)	0.0233 (2)	
O3	1.44309 (8)	0.36901 (13)	0.55130 (7)	0.0265 (2)	
O4	1.33701 (8)	0.39821 (12)	0.40099 (6)	0.0229 (2)	
O5	0.76820 (8)	0.73691 (11)	0.30121 (6)	0.0191 (2)	
O6	0.87386 (8)	0.93847 (12)	0.29701 (6)	0.0200 (2)	
N1	1.01173 (9)	0.76742 (13)	0.56858 (7)	0.0166 (2)	
H1	1.0252	0.7860	0.6283	0.020*	
N2	0.91649 (9)	0.83451 (13)	0.49714 (7)	0.0160 (2)	
N3	1.22384 (9)	0.64213 (13)	0.72129 (7)	0.0168 (2)	
N4	1.35479 (9)	0.42076 (13)	0.48519 (7)	0.0168 (2)	
N5	0.79296 (9)	0.87393 (13)	0.31434 (7)	0.0147 (2)	
C1	1.09536 (10)	0.68522 (15)	0.54842 (8)	0.0145 (2)	
C2	1.19951 (11)	0.62140 (14)	0.62097 (8)	0.0143 (2)	
C3	1.28425 (10)	0.53651 (14)	0.59994 (8)	0.0151 (2)	
H3A	1.3523	0.4966	0.6483	0.018*	
C4	1.26628 (10)	0.51219 (15)	0.50638 (8)	0.0151 (2)	
C5	1.16558 (11)	0.57308 (15)	0.43212 (8)	0.0170 (2)	
H5A	1.1555	0.5560	0.3693	0.020*	
C6	1.08201 (11)	0.65806 (16)	0.45296 (8)	0.0171 (2)	
H6A	1.0153	0.6987	0.4037	0.021*	
C7	0.83449 (11)	0.89627 (15)	0.52181 (8)	0.0152 (2)	
C8	0.73396 (10)	0.97562 (15)	0.44578 (8)	0.0156 (2)	
C9	0.71612 (10)	0.97024 (14)	0.34927 (8)	0.0143 (2)	
C10	0.62471 (11)	1.04965 (15)	0.27935 (9)	0.0182 (3)	
H10A	0.6165	1.0430	0.2163	0.022*	
C11	0.54516 (11)	1.13956 (16)	0.30456 (10)	0.0207 (3)	
H11A	0.4835	1.1942	0.2585	0.025*	
C12	0.55861 (11)	1.14699 (17)	0.39886 (10)	0.0214 (3)	
H12A	0.5048	1.2056	0.4159	0.026*	
C13	0.65176 (11)	1.06765 (16)	0.46806 (9)	0.0192 (3)	
H13A	0.6600	1.0757	0.5311	0.023*	
C14	0.83892 (12)	0.89321 (17)	0.62186 (9)	0.0212 (3)	
H14A	0.8708	0.7959	0.6507	0.032*	
H14B	0.7586	0.9067	0.6209	0.032*	
H14C	0.8902	0.9750	0.6578	0.032*	

### Atomic displacement parameters (Å^2^)

**Table:**

	*U*^11^	*U*^22^	*U*^33^	*U*^12^	*U*^13^	*U*^23^
O1	0.0217 (4)	0.0283 (6)	0.0134 (4)	0.0021 (4)	0.0035 (3)	0.0030 (4)
O2	0.0306 (5)	0.0261 (6)	0.0165 (4)	0.0090 (4)	0.0126 (4)	0.0021 (4)
O3	0.0211 (4)	0.0347 (6)	0.0202 (5)	0.0117 (4)	0.0041 (4)	0.0004 (4)
O4	0.0234 (4)	0.0294 (6)	0.0170 (4)	0.0045 (4)	0.0091 (4)	−0.0032 (4)
O5	0.0240 (4)	0.0140 (5)	0.0189 (4)	−0.0013 (4)	0.0079 (4)	−0.0031 (3)
O6	0.0177 (4)	0.0241 (5)	0.0204 (4)	−0.0010 (4)	0.0098 (3)	0.0028 (4)
N1	0.0187 (5)	0.0190 (6)	0.0121 (4)	0.0042 (4)	0.0059 (4)	0.0001 (4)
N2	0.0165 (4)	0.0168 (6)	0.0141 (4)	0.0026 (4)	0.0053 (4)	0.0002 (4)
N3	0.0218 (5)	0.0157 (6)	0.0134 (4)	0.0002 (4)	0.0074 (4)	0.0019 (4)
N4	0.0160 (4)	0.0171 (6)	0.0177 (5)	0.0008 (4)	0.0070 (4)	−0.0014 (4)
N5	0.0150 (4)	0.0171 (6)	0.0113 (4)	0.0008 (4)	0.0042 (4)	0.0006 (4)
C1	0.0166 (5)	0.0130 (6)	0.0147 (5)	−0.0006 (4)	0.0067 (4)	−0.0006 (4)
C2	0.0178 (5)	0.0138 (6)	0.0116 (5)	−0.0004 (4)	0.0060 (4)	0.0009 (4)
C3	0.0156 (5)	0.0140 (6)	0.0151 (5)	−0.0008 (4)	0.0053 (4)	0.0013 (4)
C4	0.0159 (5)	0.0146 (6)	0.0160 (5)	0.0006 (4)	0.0073 (4)	−0.0005 (4)
C5	0.0193 (5)	0.0185 (7)	0.0133 (5)	0.0008 (5)	0.0065 (4)	−0.0008 (5)
C6	0.0183 (5)	0.0192 (7)	0.0128 (5)	0.0024 (5)	0.0047 (4)	−0.0003 (5)
C7	0.0186 (5)	0.0128 (6)	0.0164 (5)	−0.0011 (4)	0.0091 (4)	−0.0011 (4)
C8	0.0155 (5)	0.0143 (6)	0.0182 (5)	−0.0016 (4)	0.0078 (4)	−0.0018 (4)
C9	0.0135 (5)	0.0119 (6)	0.0183 (5)	−0.0013 (4)	0.0070 (4)	−0.0026 (4)
C10	0.0166 (5)	0.0175 (7)	0.0182 (5)	−0.0004 (5)	0.0040 (4)	−0.0016 (5)
C11	0.0149 (5)	0.0167 (7)	0.0263 (6)	0.0011 (5)	0.0030 (5)	−0.0016 (5)
C12	0.0159 (5)	0.0193 (7)	0.0303 (7)	0.0011 (5)	0.0102 (5)	−0.0051 (5)
C13	0.0190 (5)	0.0180 (7)	0.0237 (6)	−0.0003 (5)	0.0118 (5)	−0.0035 (5)
C14	0.0239 (6)	0.0262 (8)	0.0172 (6)	0.0030 (5)	0.0121 (5)	0.0011 (5)

### Geometric parameters (Å, °)

**Table:**

O1—N3	1.2259 (13)	C5—C6	1.3710 (17)
O2—N3	1.2424 (14)	C5—H5A	0.9300
O3—N4	1.2309 (14)	C6—H6A	0.9300
O4—N4	1.2332 (13)	C7—C8	1.4807 (17)
O5—N5	1.2220 (14)	C7—C14	1.5051 (16)
O6—N5	1.2284 (13)	C8—C13	1.4029 (16)
N1—C1	1.3535 (15)	C8—C9	1.4037 (16)
N1—N2	1.3672 (14)	C9—C10	1.3824 (17)
N1—H1	0.8766	C10—C11	1.3909 (18)
N2—C7	1.2909 (15)	C10—H10A	0.9300
N3—C2	1.4539 (14)	C11—C12	1.3849 (19)
N4—C4	1.4521 (15)	C11—H11A	0.9300
N5—C9	1.4812 (15)	C12—C13	1.3874 (18)
C1—C6	1.4210 (16)	C12—H12A	0.9300
C1—C2	1.4224 (16)	C13—H13A	0.9300
C2—C3	1.3835 (16)	C14—H14A	0.9600
C3—C4	1.3744 (16)	C14—H14B	0.9600
C3—H3A	0.9300	C14—H14C	0.9600
C4—C5	1.4014 (16)		
			
C1—N1—N2	120.26 (10)	C5—C6—H6A	119.4
C1—N1—H1	118.2	C1—C6—H6A	119.4
N2—N1—H1	121.0	N2—C7—C8	116.26 (10)
C7—N2—N1	115.88 (10)	N2—C7—C14	123.38 (11)
O1—N3—O2	122.46 (10)	C8—C7—C14	120.34 (10)
O1—N3—C2	118.58 (10)	C13—C8—C9	115.60 (11)
O2—N3—C2	118.96 (10)	C13—C8—C7	120.47 (11)
O3—N4—O4	123.16 (10)	C9—C8—C7	123.89 (11)
O3—N4—C4	119.00 (10)	C10—C9—C8	123.37 (11)
O4—N4—C4	117.84 (10)	C10—C9—N5	114.72 (10)
O5—N5—O6	124.63 (10)	C8—C9—N5	121.88 (10)
O5—N5—C9	117.57 (10)	C9—C10—C11	119.14 (12)
O6—N5—C9	117.73 (10)	C9—C10—H10A	120.4
N1—C1—C6	121.03 (11)	C11—C10—H10A	120.4
N1—C1—C2	121.97 (10)	C12—C11—C10	119.46 (12)
C6—C1—C2	117.01 (11)	C12—C11—H11A	120.3
C3—C2—C1	121.72 (10)	C10—C11—H11A	120.3
C3—C2—N3	116.05 (10)	C11—C12—C13	120.47 (12)
C1—C2—N3	122.23 (10)	C11—C12—H12A	119.8
C4—C3—C2	118.92 (11)	C13—C12—H12A	119.8
C4—C3—H3A	120.5	C12—C13—C8	121.95 (12)
C2—C3—H3A	120.5	C12—C13—H13A	119.0
C3—C4—C5	121.71 (11)	C8—C13—H13A	119.0
C3—C4—N4	118.41 (10)	C7—C14—H14A	109.5
C5—C4—N4	119.88 (10)	C7—C14—H14B	109.5
C6—C5—C4	119.37 (11)	H14A—C14—H14B	109.5
C6—C5—H5A	120.3	C7—C14—H14C	109.5
C4—C5—H5A	120.3	H14A—C14—H14C	109.5
C5—C6—C1	121.27 (11)	H14B—C14—H14C	109.5
			
C1—N1—N2—C7	172.81 (11)	C2—C1—C6—C5	0.48 (19)
N2—N1—C1—C6	−4.25 (18)	N1—N2—C7—C8	177.22 (10)
N2—N1—C1—C2	176.20 (11)	N1—N2—C7—C14	−1.52 (18)
N1—C1—C2—C3	179.52 (11)	N2—C7—C8—C13	−169.40 (12)
C6—C1—C2—C3	−0.04 (18)	C14—C7—C8—C13	9.39 (18)
N1—C1—C2—N3	−0.93 (19)	N2—C7—C8—C9	8.14 (18)
C6—C1—C2—N3	179.51 (11)	C14—C7—C8—C9	−173.07 (12)
O1—N3—C2—C3	7.08 (17)	C13—C8—C9—C10	0.25 (18)
O2—N3—C2—C3	−172.76 (11)	C7—C8—C9—C10	−177.40 (12)
O1—N3—C2—C1	−172.51 (12)	C13—C8—C9—N5	−177.90 (11)
O2—N3—C2—C1	7.65 (18)	C7—C8—C9—N5	4.45 (18)
C1—C2—C3—C4	−0.68 (19)	O5—N5—C9—C10	−96.72 (13)
N3—C2—C3—C4	179.74 (11)	O6—N5—C9—C10	80.43 (13)
C2—C3—C4—C5	1.00 (19)	O5—N5—C9—C8	81.58 (14)
C2—C3—C4—N4	−178.99 (11)	O6—N5—C9—C8	−101.27 (13)
O3—N4—C4—C3	−0.47 (18)	C8—C9—C10—C11	−0.28 (19)
O4—N4—C4—C3	179.19 (11)	N5—C9—C10—C11	177.99 (11)
O3—N4—C4—C5	179.54 (12)	C9—C10—C11—C12	−0.39 (19)
O4—N4—C4—C5	−0.81 (17)	C10—C11—C12—C13	1.1 (2)
C3—C4—C5—C6	−0.6 (2)	C11—C12—C13—C8	−1.1 (2)
N4—C4—C5—C6	179.41 (12)	C9—C8—C13—C12	0.45 (18)
C4—C5—C6—C1	−0.2 (2)	C7—C8—C13—C12	178.18 (12)
N1—C1—C6—C5	−179.09 (12)		

### Hydrogen-bond geometry (Å, °)

**Table:**

*D*—H···*A*	*D*—H	H···*A*	*D*···*A*	*D*—H···*A*
N1—H1···O2	0.88	1.94	2.6026 (13)	131
C10—H10A···O4^i^	0.93	2.42	3.2313 (16)	146
C12—H12A···O4^ii^	0.93	2.55	3.4353 (18)	159

Symmetry codes:  (i) −*x*+2, *y*+1/2, −*z*+1/2; (ii) *x*−1, *y*+1, *z*.

## References

[bb1] Allen, F. H., Kennard, O., Watson, D. G., Brammer, L., Orpen, A. G. & Taylor, R. (1987). *J. Chem. Soc. Perkin Trans. 2*, pp. S1–19.

[bb2] Bendre, R., Murugkar, A., Padhye, S., Kulkarni, P. & Karve, M. (1998). *Met. Based Drugs*, **5**, 59–66. 10.1155/MBD.1998.59 PMC2365098 18475824

[bb3] Bernstein, J., Davis, R. E., Shimoni, L. & Chang, N.-L. (1995). *Angew. Chem. Int. Ed. Engl.* **34**, 1555–1573.

[bb4] Bruker (2005). *APEX2*, *SAINT* and *SADABS* Bruker AXS Inc., Madison, Wisconsin, USA.

[bb5] Cosier, J. & Glazer, A. M. (1986). *J. Appl. Cryst.* **19**, 105–107.

[bb6] Fun, H.-K., Jansrisewangwong, P. & Chantrapromma, S. (2011). *Acta Cryst.* E**67**, o1034–o1035. 10.1107/S1600536811011135 PMC3089201 21754363

[bb7] Nakamura, A. & Goto, S. (1996). *J. Biochem.* **119**, 768–774. 10.1093/oxfordjournals.jbchem.a021306 8743580

[bb8] Rollas, S. & Küçükgüzel, S. G. (2007). *Molecules*, **12**, 1910–1939. 10.3390/12081910 PMC6149174 17960096

[bb9] Shan, S., Xu, D.-J., Hung, C.-H., Wu, J.-Y. & Chiang, M. Y. (2003). *Acta Cryst.* C**59**, o135–o136. 10.1107/s0108270103002464 12711787

[bb10] Sheldrick, G. M. (2008). *Acta Cryst.* A**64**, 112–122. 10.1107/S0108767307043930 18156677

[bb11] Singh, K. S., Mozharivskyj, Y. A., Thöne, C. & Kollipara, M. R. (2005). *J. Organomet. Chem.* **690**, 3720–3729.

[bb12] Spek, A. L. (2009). *Acta Cryst.* D**65**, 148–155. 10.1107/S090744490804362X PMC2631630 19171970

[bb13] Yacorb, Y. (1999). *Proc. IMechE Part D J. Automobile Eng.* **213**, 503–517.

